# Mixed depression and childhood trauma in individuals with bipolar disorders

**DOI:** 10.1192/j.eurpsy.2026.11213

**Published:** 2026-07-31

**Authors:** F. Bardi, M. Pinto, S. Montanari, C. Calderoni, G. Sani, D. Janiri

**Affiliations:** 1 Section of Psychiatry, Department of Neuroscience, Head-Neck and Chest, Università Cattolica del Sacro Cuore; 2 Section of Psychiatry, Department of Neuroscience, Head-Neck and Chest, Fondazione Policlinico Universitario Agostino Gemelli IRCCS, Rome, Italy

## Abstract

**Introduction:**

Mixed Depression (MxD), characterized by the co-occurrence of depressive and excitatory symptoms, is a frequent yet often underdiagnosed manifestation of bipolar disorders (BDs), associated with poorer prognosis and treatment outcomes. Childhood trauma (CT) is another major factor linked to greater illness severity in BDs. However, no studies have directly examined the association between CT and MxD in BD individuals.

**Objectives:**

To investigate the relationship between childhood trauma and the lifetime tendency to present mixed features during depressive episodes in bipolar disorder.

**Methods:**

A total of 220 euthymic BD outpatients and 156 healthy controls (HCs) were enrolled. BD patients underwent a semistructured interview and retrospective assessment of depressive episodes, and were classified according to Koukopoulos’ criteria as MxD (n=100) or non-MxD (n=120). All participants completed the Childhood Trauma Questionnaire (CTQ). Group comparisons were conducted via ANOVAs/MANOVAs, and logistic regressions tested the predictive role of CT subtypes for MxD.

**Results:**

Significant group differences in CT emerged (p<0.001). MxD patients showed higher CTQ total scores than both non-MxD (p=0.029) and HCs (p<0.001). Emotional abuse was particularly elevated in MxD vs. non-MxD (p<0.001). Compared with HCs, the MxD group reported higher emotional abuse, emotional neglect, and physical abuse (all p<0.001), while non-MxD patients showed higher physical abuse and neglect (p<0.01). Higher CT scores significantly predicted MxD group membership.

**Conclusions:**

Individuals with BD report higher CT levels than the general population. Importantly, emotional abuse appears specifically associated with mixed depressive features, suggesting a role in the complexity and instability of mood presentations in BD. Conversely, physical trauma may reflect a broader vulnerability to mood dysregulation rather than to the mixed phenotype itself.

**Disclosure of Interest:**

None Declared

